# Words for the Wise

**Published:** 1887-05-28

**Authors:** 


### WORDS FOR THE WISE.

The healing art can boast no home
More fraught with zeal and knowledge
Than that whose shadow sweeps the dome
Of University College.
She, midst a goodly sister band,
Though young, yet for rebuilding
Bespeaks a widely opened hand—
Not for mere paint and gilding.

Aright to spread her garnered love,
To cope with deadly scourges,
More liberal housing needs, much more—
So keen experience urges—
Of kindly-tempered, purest air,
With cheerful light, unswerving—
Unstinted be your help ; nor spare
Till aid o'ertakes deserving !—OSTRICH.

"Good wine needs no bush :" and a hospital of all institutions least needs persuasive advocacy. All know what illness is. Some have been ill themselves, and know by experience either the need or the value of good advice and nursing. Some have seen others suffering from illness or accident : and these owe a debt of gratitude for their inexperience, which they may pay in part by a liberal gift to the hospital.—H. B.

